# Nanophytosome-functionalized active packaging films for preservation of refrigerated rainbow trout

**DOI:** 10.1016/j.fochx.2025.102708

**Published:** 2025-06-28

**Authors:** Mohammad Maleki, Mahmood Alizadeh Sani, Roya Rezaeian-Doloei, David Julian McClements, Mohammad Mohsenzadeh

**Affiliations:** a Department of Food Hygiene and Aquaculture, Faculty of Veterinary Medicine, Ferdowsi University of Mashhad, Mashhad, Iran; b Department of Food Science and Technology, School of Nutritional Sciences and Dietetics, Tehran University of Medical Sciences, Tehran, Iran; c Department of Agricultural sciences, Mashhad Branch, Islamic Azad University, Mashhad, Iran; d Department of Agricultural sciences, Arid Environments Research center, Mashhad Branch, Islamic Azad University, Mashhad, Iran; e Department of Food Science, University of Massachusetts Amherst, Amherst, MA 01003, USA

**Keywords:** Antimicrobial agents, Nanotechnology, Polymers, Nanophytosome, particle size

## Abstract

The application of nanophytosomes in active packaging is an exciting area of research and innovation within food packaging and preservation technologies. Nanophytosomes, which are nanoscale carriers derived from plant extracts, offer several unique properties that enhance the performance of active packaging systems. In this study, nanophytosomes of *Perovskia abrotanoides* Kar. essential oil (PEO-NP; 10, 20, and 30 mg) and catechin (C-NP; 2.5, 5, and 10 mg) were prepared using the thin layer hydration method. As the nanophytosome concentration increased, the particle size decreased, while the encapsulation efficiency and loading capacity increased. At sufficiently high concentrations, bioactive-loaded nanophytosomes with small mean particle diameters (< 200 nm), low polydispersity indices (≤ 0.2), and negative charges (−34 to −52 mV) could be produced. Transmission electron microscopy images showed that the nanophytosomes were small spherical particles. Encapsulation of the essential oil and catechin within the nanophytosomes improved their stability under refrigerated storage and gastrointestinal conditions. Moreover, the nanophytosome-encapsulated bioactives exhibited greater antioxidant and antibacterial effects than the free ones. Finally, incorporating the bioactive-loaded nanophytosomes into active packaging materials was shown to extend the shelf life of fish fillets, which was attributed to their ability to suppress microbial growth and oxidation. The nanophytosomes developed in this study may therefore be suitable for improving the performance of plant-based preservatives within food and other industries. The incorporation of nanophytosomes into active packaging represents a significant increase forward in food preservation technology. By enhancing delivery mechanisms, improving material properties, and leveraging natural bioactive compounds, nanophytosomes can revolutionize the way food products are packaged and preserved, responding to both market demands and sustainability goals. Further research and development are essential for overcoming challenges related to scalability, regulatory approval, and consumer acceptance.

## Highlights

• Nanophytosomes loaded with either essential oils or catechin were prepared.
• These nanophytosomes were small, spherical, anionic particles.
• The bioactive-loaded phytosomes exhibited great functional properties.
• Catechin-loaded phytosomes proved to be more effective than essential oil.
• Packaging films containing nanophytosomes increased the shelf life of fish.

## 1 Introduction

The shelf life and safety of foods can be extended by adding synthetic or natural preservatives that inhibit the growth of spoilage or pathogenic microorganisms, as well as retarding undesirable chemical reactions, such as oxidation (Sani, Khezerlou, & McClements, 2024; Sani, Khezerlou, Tavassoli, et al., 2024; Teshome et al., 2022). Recently, there has been a growing demand for natural preservatives due to consumer concerns about the adverse effects of synthetic ones on human health and the environment (Sani et al., 2023, Sani et al. 2024). Researchers have identified numerous kinds of natural substances that exhibit antimicrobial and/or antioxidant activities, including plant extracts, such as essential oils and phytochemicals (Gyawali & Ibrahim, 2014). However, many of these plant extracts have limited application in foods because of their low water solubility, poor chemical stability, and/or low bioactivity (Dias et al., 2015; Sani et al., 2022a; Sani et al., 2022b).

These challenges can often be overcome by encapsulating the plant extracts within suitable nanoparticle-based delivery systems (Amiri et al., 2018). Nanophytosomes are phospholipid-based vesicular delivery systems that have been used to improve the dispersibility, stability, and efficacy of bioactive compounds (Mancini et al., 2018). Indeed, previous studies have already shown that nanophytosomes can enhance the stability of encapsulated substances during simulated food processing and storage (Babazadeh et al., 2018; Huang et al., 2020). Typically, these substances are held inside the nanophytosomes by physical interactions, such as van der Waals, hydrogen bonding, hydrophobic, and electrostatic interactions. Hydrophobic substances are usually located within the hydrophobic domains formed by the non-polar tails of the phospholipids, whereas hydrophilic substances are usually located within the aqueous core or within the hydrophilic domains formed by the polar head groups of the phospholipids (Vu et al., 2018).

Existing nanocarrier systems often struggle with food preservation due to limitations in stability, controlled release, and bioavailability of active compounds. They may not effectively protect sensitive ingredients from environmental factors or ensure sustained effectiveness over time. Many traditional nanocarriers, such as liposomes or solid lipid nanoparticles, can be prone to aggregation or degradation under varying environmental conditions (light, heat, and moisture). This instability can compromise their ability to protect active ingredients in food applications (Sani et al., 2022a; Sani et al., 2022b). Conventional nanocarriers often lack or have less ability to modulate the release of encapsulated compounds effectively. In food applications, a controlled release is crucial to ensure that preservatives or antioxidants are available throughout the product's shelf life, preventing spoilage. Some nanocarrier systems may not promote optimal absorption of bioactive compounds within the food matrix. Poor bioavailability can limit the effectiveness of preservatives or nutrients designed to enhance food safety and longevity. Traditional nanocarriers may interact negatively with other food components, affecting their performance and resulting in undesirable changes to flavor, texture, or nutritional quality (Mohammadi et al., 2022).

In contrast, nanophytosomes are particularly designed to encapsulate plant-based bioactives, which can offer greater resistance to environmental factors. Their unique structure helps protect sensitive compounds from degradation. Nanophytosomes offer enhanced encapsulation and stability, improving the delivery of bioactive compounds while allowing for targeted release. Nanophytosomes allow for more precise control over the release of active ingredients, which can significantly improve their efficacy in preventing spoilage. This makes them more suitable for preserving food quality and extending shelf life (Ghanbarzadeh et al., 2016a; Ghanbarzadeh et al., 2016b; Khosh Manzar et al., 2021b).

In summary, while existing nanocarrier systems have certain advantages, they often cannot provide the optimal combination of stability, controlled release, and bioavailability necessary for effective food preservation. Nanophytosomes address these challenges, making them a more promising solution for enhancing food quality and extending shelf life. This distinctive ability positions them ahead in applications that rely on advanced preservation techniques, especially in food packaging applications.

A broad range of plant-derived substances may be utilized as preservatives in foods. These substances are often produced by plants to protect themselves from stresses in their environments (Beya et al., 2021). For instance, many of the phenolic compounds naturally produced by plants are secondary metabolites that exhibit antioxidant and antimicrobial activities (Manzoor et al., 2023). Catechin (C) is a phenolic compound found in many fruits, vegetables, and herbs (Grzesik et al., 2018; Jiang et al., 2021), which has been reported to exhibit both antioxidant and antibacterial activities (Aree & Jongrungruangchok, 2016). However, catechin is susceptible to degradation when exposed to certain environmental stresses (such as high oxygen, light, temperature, and alkaline conditions), which reduces its bioactivity (Maghsoudlou et al., 2019; Sabaghi et al., 2020). We hypothesized that encapsulating catechin within nanophytosomes would protect it from degradation when exposed to these kinds of environmental stresses. This hypothesis is based on the findings of previous studies, which have shown that nanoencapsulation can increase the stability and bioactivity of phenolic substances (Guía-García et al., 2022; Jiang et al., 2021; Pateiro et al., 2021).

Nanophytosomes offer a unique approach to active packaging by utilizing natural plant extracts, which provide high encapsulation efficiency and biocompatibility, making them appealing to health-conscious consumers. They facilitate the controlled release of bioactive compounds, enhancing food shelf life and safety while posing fewer toxicity risks compared to synthetic carriers like liposomes and polymeric nanoparticles. Although nanophytosomes can be cost-effective, challenges in large-scale production remain. In contrast, other carriers may require complex processes and modifications to achieve similar functional properties. Overall, nanophytosomes represent a promising innovation in active packaging technologies, balancing safety, efficacy, and market demands (Barani et al. Khosh Manzar et al., 2021b, Deniz et al., 2024, Kamireddy et al., 2024).

Plant essential oils (EOs) are oily substances extracted from various plants using physical and/or chemical methods (Ni et al., 2021). These oils typically contain a broad spectrum of bioactive compounds that exhibit antioxidant and/or antimicrobial properties (Bolouri et al., 2022; Liu et al., 2014). In this study, we focused on essential oils extracted from *Perovskia abrotanoides,* which is a fragrant and aromatic pltio2ant (sometimes called “Russian sage”) that is native to Central Asia, including Iran, Pakistan, Afghanistan, Turkmenistan, and Uzbekistan (Mohammadhosseini et al., 2021). The *Perovskia abrotanoides* essential oil (PEO) was selected for this study because it has previously been reported to exhibit potent antioxidant and antimicrobial activities (Ahmadi & Mohsenzadeh, 2023; Ghaffari et al., 2018), which makes it particularly suitable as a natural preservative in food and beverage applications. However, PEO is a predominantly hydrophobic molecule that has poor water solubility and chemical stability. For this reason, we hypothesized that encapsulating PEO in nanophytosomes would improve its efficacy.

The purpose of this study was to optimize the formulation of nanophytosomes containing catechin or PEO, as well as to compare their preservative properties. Initially, the impact of incorporating different levels of these bioactive substances into the nanophytosomes on their physicochemical, structural, and encapsulation properties was studied. Then, the antioxidant and antibacterial properties of the catechin-loaded (C-NPs) and PEO-loaded nanophytosomes (PEO-NPs) were evaluated. This research aims to identify the optimal conditions to produce these plant-based preservatives, as well as to evaluate their potential efficacy. In particular, the effectiveness of nanophytosome-loaded active packaging materials in extending the shelf life of fish was investigated by analyzing changes in their chemical and microbial properties during storage. The results of microbial and chemical tests showed that the use of phytosomes can significantly increase the shelf life of packaged rainbow trout compared to the control sample. These features demonstrate the importance and innovative aspects of phytosomes and their application in the packaging industry. As a result, it reduces the need to use chemical additives as preservatives and ensures consumer safety and food products.

## 2 Materials and methods

### 2.1 Materials

Lecithin (purity >99 %), catechin (purity >98 %), carboxymethyl cellulose, glycerol, and other reagents were procured from the Merck company (Germany). DPPH standard was purchased from Sigma-Aldrich (USA). *Perovskia abrotanoides* essential oil (PEO) was purchased from Johare Taem Shargh (Mashhad, Iran). The bacterial strains *Escherichia coli* ACCC 10141, *Bacillus subtilis* ACCC 10242, *Staphylococcus aureus* ATCC 25923 bacteria were obtained from the microbial collection of the Faculty of Veterinary Medicine, Ferdowsi University of Mashhad, Iran. All materials used in this study were analytical grade.

### 2.2 Preparation of Nanophytosomes (NPs)

The preparation of PEO-NPs and C-NPs was carried out using a thin layer hydration method described previously with slight modifications, without using different ratios of lecithin to bioactive substance (PEO and catechin) (Khosh manzar et al., 2021). Nanophytosomes were prepared that contained different concentrations of either catechin (2.5, 5, and 10 mg) or PEO (10, 20, and 30 mg) using 60 mg of lecithin (selected based on pre-laboratory tests and previous studies) (Khosh manzar et al. Khosh Manzar et al., 2021b). The ingredients were mixed using a stirrer in 10 mL of 96 % ethanol for 30 min. The solution was then held for 12 h at 4 °C and then the ethanol was evaporated under vacuum using a rotary evaporator at 45 °C. To form the nanophytosome particles, 10 mL of deionized water was added to the dried film layer, and an ultrasound probe (model UP200H, Heilscher, Germany; 200 W, 24 kHz with a titanium microtip of 3 mm diameter) was used to sonicate the samples (10 cycles: 1 min on/1 min off) at an amplitude of 80 W.

### 2.3 Characterization of NPs

***Particle characteristics:*** A combined dynamic light scattering/particle electrophoresis instrument (SZ100 Model, Horiba, Japan) was used to determine the mean particle size, polydispersity index (PDI), and zeta potential of the PEO-NPs and C-NPs. The samples were diluted in a buffer solution before analysis.

***Morphology**:* Transmission electron microscopy (TEM, CM120 model, Netherlands) was used to provide information about the microstructure of the nanophytosomes. Samples were diluted with ethanol at a ratio of 1:20 and sonicated for 10 min. A drop of phytosome was placed onto a carbon-coated grid, allowing to create a thin film, and the phytosome images were captured using TEM.

***Encapsulation efficiency and loading capacity**:* The encapsulation efficiency (EE) and loading capacity (LC) of the nanophytosomes was determined using the methods described previously (Nazari, Ghanbarzadeh, et al., 2019; Ahmad et al., 2019). Initially, a calibration curve was prepared by measuring the absorbance versus concentration profile of each phytochemical using a UV–visible spectrophotometer (CAMSPEC M550, United Kingdom). An appropriate amount of nanophytosome dispersion (50 mg/ 10 mL ethanol) was introduced into the upper part of a Millipore Amicon® Ultra filtration tube and centrifuged at 900 rpm for 5 min. The levels of PEO and catechin were evaluated by recording the absorbance at λ_max_ = 217 nm and 280 nm respectively, utilizing the calibration curve within a concentration range of 1–20 μg/mL (*n* = 5) with a spectrophotometer.

***Free radical scavenging activity***: The free radical scavenging activity of the PEO-NPs, C-NPs, and free nanophytosomes was evaluated using the DPPH method (Nazari, Ghanbarzadeh, et al., 2019). In this method, different concentrations of nanophytosomes were prepared in distilled water. After 1 and 30 days, 1 mL of sample was mixed with 1 mL of DPPH solution (0.004 %) and the mixture was stored for 5 h in a dark room at room temperature. The absorbance values of the samples were then measured and compared to those of a DPPH standard solution at a wavelength of 517 nm using a UV–Vis spectrophotometer (Thermo Biomate 5 model, The Lab World Group, Boston, USA)

***Turbidity**:* The turbidity of the PEO-NP and C-NP dispersions was measured at a wavelength of 600 nm using a UV–visible spectrophotometry (Thermo Biomate 5 model, The Lab World Group, Boston, USA) method described previously (Amiri et al., 2018).

***Physical stability**:* The stability of the nanophytosomes was determined by storing their aqueous dispersions at room temperature (25 °C) for one month and then measuring the particle size, polydispersity, zeta potential, EE, and LC of the samples using the methods described earlier.

***FTIR analysis:*** Information about the nature of the functional groups present and their interactions was obtained by measuring their infrared spectra using a Fourier transform infrared spectroscopy (Thermo Nicolet Avatar 380 FTIR, Thermo Electron Scientific Instruments Corporation, Madison, WI, USA) spectrometer from 400 to 4000 cm^−1^ (Khosh manzar et al., 2021; Ahmad et al., 2019).

***Gastrointestinal stability**:* The stability of the catechin and PEO encapsulated within the nanophytosomes when incubated in simulated gastric fluids (SGFs) and simulated intestinal fluids (SIFs) was investigated using a spectrophotometer method (Abdulqahar et al., 2022). The SGFs were prepared by mixing 7 mL hydrochloric acid, 2 g sodium chloride, and 3.2 mg/mL of pepsin, and then adjusting the volume to 1 L using distilled water. The final pH of this solution was then adjusted to 1.2 using 1 N hydrochloric acid. The SIFs were prepared by mixing 3.2 mg/mL of pancreatin, 6.8 g of potassium hydrogen phosphate, 200 mL of sodium hydroxide (0.1 M), and 5.16 mg/mL bile salts in distilled water and adjusting to pH 7.0. For each test, two millimeters of sample were incubated in a shaking water bath at 37 °C and tested at time intervals of 0.5, 1, 1.5, 2, 2.5, 3, 3.5 and 4 h (Khosh manzar et al., 2021).

***Antibacterial activity***: The antibacterial activity of the PEO-NPs and C-NPs against model pathogenic bacteria, *Staphylococcus aureus* (ATCC 25923), *Bacillus subtilis* (ATCC 6633) and *Escherichia coli* (ATCC 25922), was investigated using an agar well-diffusion (diameter = 7 mm) antimicrobial assay, according to a method described previously (Li et al., 2018).

### 2.4 Application of active packaging films for rainbow trout preservation

#### 2.4.1 Preparation of active packaging films

Films were prepared according to a method described previously (Maleki & Mohsenzadeh, 2022). Initially, carboxymethyl cellulose (CMC) (1.5 g) was mixed with 100 mL of distilled water at 60 °C for 15 min. Glycerol (40 % by weight of dry matter) was then added and the solution was mixed for 10 min. Then 10 mg of catechin nanophytosome (T1) and 30 mg of essential oil nanophytosome (T2) were added to the CMC solution separately and mixed for 15 min. In the end, the film-forming solution was dried using the casting method by placing it in an oven for 24 h at 30 °C.

#### 2.4.2 Rainbow trout samples and treatment preparation

Rainbow Trout was procured from a local market (Mashhad, Iran) and transported to the laboratory under cold/hygienic conditions. The fish fillet was divided into pieces of about 50 g with an average thickness of 2 cm. The fish pieces were covered with active films (T1 and T2 films), while the control (C) samples were simply packed in sterile plastic bags. Then, fish samples were kept at a cold temperature (4 °C ± 1). All samples were characterized using chemical assays (pH, thiobarbituric acid (TBA), and total volatile basic nitrogen (TVB-N)), as well as microbiological assays (psychrotrophic bacteria count (PBC), and total viable count (TVC)) at intervals of 0, 3, 6, 9 and 12 days (Alizadeh-Sani et al., 2020).

##### 2.4.2.1 Chemical analysis

***pH:*** Each sample (10 g) was homogenized in 100 mL of distilled water and then filtered. A digital pH meter was then used to measure the pH of the resulting sample solution (Alizadeh-Sani et al., 2020).

***TBA:*** The thiobarbituric acid concentration in the samples was determined using a spectrophotometer method and expressed as mg MDA/kg sample. Briefly, minced fish samples (4 g) were dissolved in trichloroacetic acid (TCA) (20 % *w*/*v*) to a volume of 20 mL and then mixed at 15,000 rpm for 2 min. Then, 3 mL of the solution was mixed with 3 mL of 2-trichloroacetic acid (0.1 % w/v) and kept at 95 °C for 2 h, and then the absorbance was recorded at 532 nm (Alizadeh-Sani et al., 2020; Yin et al., 2022).

***TVB-N:*** Samples (10 g) were mixed with distilled water (300 mL) and MgO (2 g) and then distilled. The liquid from the distillation was collected in an Erlenmeyer flask (250 mL) containing boric acid (25 mL, 2 %) and methyl red (0.04 mL) for titration with ammonia until the solution turned green and alkaline, indicating the formation of TVB-N. This boric acid solution was then titrated with 0.1 N HCl until the appearance of a pink color was observed. The TVB-N content (mg N/100 g) was obtained based on the volume of HCl used for titration (Alizadeh-Sani et al., 2020; Yin et al., 2022).

##### 2.4.2.2 Microbial analysis

***Total viable count (TVC):*** Fish samples (10 g) were transferred to 90 mL of 0.1 % peptone water and blended with a mixer (Lab Blender 400, Stomacher, USA) for 1 min at room temperature. Then, 0.1 % peptone water was used to prepare different dilutions and TVC was enumerated on a nutrient agar medium after incubation at 37 °C for 48 h (Alizadeh-Sani et al., 2020; Yin et al., 2022).

***Psychrotrophic bacteria count (PBC):*** Fish samples (10 g) were transferred to 90 mL of 0.1 % peptone water and blended with a mixer (Lab Blender 400, Stomacher, USA) for 1 min at room temperature. Then, 0.1 % peptone water was used to prepare different dilutions and the PBC enumerated on tryptic soy agar (TSA) after incubation at 7 °C for 10 days (Alizadeh-Sani et al., 2020; Yin et al., 2022).

### 2.5 Statistical analysis

Statistical analysis was carried out using SPSS software (version 16, SPSS, Inc. Chicago, IL, USA), employing one-way analysis of variance with multiple comparisons between treatments, followed by Duncan's test at a significance level of 5 %. The analysis was performed in triplicate for all treatments.

## 3 Results and discussion

### 3.1 Nanophytosome characteristics

Initially, the physicochemical and structural properties of nanophytosomes containing different bioactive types and concentrations were characterized.

***Particle characteristics:*** The mean particle diameters of C-NPs containing 2.5, 5, or 10 mg of catechin were 162, 178, and 181 nm, while those of the PEO-NPs containing 10, 20, or 30 mg of essential oil were 140, 153, and 166 nm, respectively. These results show that bioactive-loaded nanophytosomes with relatively small dimensions could be successfully prepared using the thin layer-hydration-sonication fabrication method employed in this study. The particle sizes of the C-NPs and PEO-NPs measured in our study are consistent with those reported in previous studies for epigallocatechin-3-gallate-loaded nanophytosomes (Shariare et al., 2020) and garlic essential oil-loaded nanophytosomes (Nazari, Ghanbarzadeh, Kafil, et al., 2019). The mean particle diameter of both kinds of nanophytosomes increased as the bioactive concentration increased, which may have been because they swelled as they incorporated more substances (Khosh Manzar et al., 2021a; Khosh Manzar et al., 2021b). The polydispersity index (PDI) values for the C-NPs containing 2.5, 5, or 10 mg of catechin were 0.20, 0.20, and 0.19, while the PDI values for the PEO-NPs containing 10, 20, or 30 mg of essential oil were 0.18, 0.17, and 0.17, respectively. These results suggest that all the nanophytosomes had relatively narrow particle size distributions (Rafiee et al., 2017), irrespective of the amount of bioactive substance incorporated. Similar results have been reported for epigallocatechin-3-gallate-loaded nanophytosomes (Shariare et al., 2020) and for cinnamon essential oil-loaded nanophytosomes (Nazari, Ghanbarzadeh, Kafil, et al., 2019).

The electrical characteristics of nanophytosomes were characterized by measuring their zeta-potential values. Typically, nanoparticles with a relatively high absolute zeta potential value (> 30 mV) have high resistance to aggregation because of the strong electrostatic repulsion between them (Schäfer et al., 2010; Zou et al., 2014). The electrical characteristics of nanoparticles also impacts their ability to interact with the surfaces of bacteria, which can alter their antimicrobial activity (Hanpanich et al., 2017). The zeta potential values of the C-NPs containing 2.5, 5, or 10 mg of catechin were − 45, −48, and − 49 mV, while those of the PEO-NP formulations containing 10, 20, or 30 mg of essential oil were − 52, −47, and − 34 mV, respectively (Table 1). Interestingly, the zeta-potential became more negative as the concentration of catechin in the nanophytosomes increased, but less negative as the concentration of essential oil increased. These results suggest that the two different kinds of bioactive agent had different effects on their electrical characteristics of the nanophytosome surfaces.

**Table 1:** Mean particle diameter (Z average), polydispersity index (PDI), zeta-potential, turbidity, encapsulation efficiency (EE), and loading capacity (LC) of C-NP and PEO-NP dispersions.

Turbidity (600 nm)	LC (%)	EE (%)	Zeta Potential (mV)	PDI	Z average (nm)	Treatments	Nanophytosome (NPs)
0.20 ± 0.05 ^a^	70 ± 2^a^	87 ± 1^a^	−49 ± 4 ^a^	0.2 ± 0.07 ^a^	162 ± 12^a^	C-NP-1	Catechin nanophytosome (C-NP)
0.18 ± 0.04^b^	73 ± 2^b^	90 ± 1^b^	−48 ± 5 ^a^	0.2 ± 0.08 ^a^	178 ± 12^ab^	C-NP-2	
0.17 ± 0.05 ^c^	78 ± 1^c^	93 ± 2^c^	−45 ± 6 ^b^	0.19 ± 0.05 ^b^	181 ± 11^c^	C-NP-3	
0.16 ± 0.04 ^a^	62 ± 1^a^	75 ± 1^a^	−52 ± 5 ^a^	0.18 ± 0.06^a^	140 ± 11 ^a^	PEO-NP-1	*Perovskia abrotanoides* essential oil (PEO) nanophytosome (PEO-NP)
0.16 ± 0.04 ^a^	68 ± 1^b^	80 ± 3^b^	−47 ± 4 ^b^	0.17 ± 0.05 ^b^	153 ± 12 ^b^	PEO-NP-2	
0.15 ± 0.04 ^b^	74 ± 1^c^	84 ± 2^c^	−34 ± 4 ^c^	0.17 ± 0.05 ^b^	166 ± 10^c^	PEO-NP-3	

Nanophytosomes are lipid-based nanoparticles designed to enhance the bioavailability of bioactive compounds, particularly plant extracts and bioactives. They are composed of phospholipids (such as phosphatidylcholine) that form vesicles, encapsulating the desired compounds. Catechin and negative charge increase: Catechin is a flavonoid containing multiple hydroxyl (-OH) groups, which are polar and interact readily with water and phospholipid head groups. At physiological pH, some -OH groups partially deprotonate, forming negatively charged phenolate ions and increasing the overall negative surface charge of the phytosome (Khosh Manzar et al., 2021b; Ma et al., 2025).

Catechin can integrate into the phospholipid bilayer near the polar head groups. The negatively charged phenolate ions enhance electrostatic repulsion between phytosomes, increasing stability and preventing aggregation. The increased negative charge enhances colloidal stability through electrostatic repulsion, affects interactions with biological membranes, and may improve cellular uptake and hydration of the phytosome (Abdelbari & Mosallam, 2025). In other words, catechins, which are polyphenolic compounds found in various plants can contribute to an increase in the negative charge of nanophytosomes due to their chemical structure and interaction with the lipid bilayer. The increased negative charge likely results from catechins acting as anionic surfactants. These molecules can interact with the lipid components and promote a negative surface charge through adsorption or by changing the membrane's properties, which enhances their stability and dispersibility (Natarajan et al., 2019; Shariare et al., 2020).

Essential oils consist mainly of volatile, hydrophobic compounds like terpenes. These can insert into the hydrophobic core of the phospholipid bilayer, displacing water molecules and shielding negatively charged phosphate groups. This reduces the phytosome's overall negative charge. Essential oils can also disrupt phospholipid packing, increasing bilayer fluidity and altering charge accessibility (Albash et al., 2023). A reduced negative charge may decrease electrostatic repulsion, potentially impacting colloidal stability. However, increased bilayer fluidity could enhance the release of encapsulated compounds and improve interactions with biological membranes due to heightened hydrophobicity (Khosh Manzar et al., 2021b). In other words, essential oils, which contain various hydrophobic compounds, can reduce the negative charge in nanophytosomes. This reduction can occur because the hydrophobic components of essential oils may integrate into the lipid bilayer of the nanophytosome, altering its surface characteristics. The hydrophobic interaction could mask the negative charges or disrupt the arrangement of charged groups on the surface, leading to a more neutral or less negatively charged structure (Hendawy et al., 2023; Nazari, Ghanbarzadeh, Kafil, et al., 2019; Nazari, Ghanbarzadeh, Samadi Kafil, et al., 2019; Nazari, Majdi, Milani, et al., 2019).

However, molecular interactions and vibrational aspects can also affect the particle characteristics of phytosomes. For example, -OH Vibrations: The -OH groups of catechin and polar head groups of phospholipids participate in hydrogen bonding. The presence of essential oils can alter the vibrational frequencies of these -OH bonds, detectable through Fourier-transform infrared spectroscopy (FTIR). Changes in -OH vibrations indicate shifts in hydration and hydrogen bonding networks within the phytosome (Khosh Manzar et al., 2021b; Visht & Sirwan Salih, 2023).

-C=C Vibrations: Catechin and essential oil components feature C=C double bonds. Their vibrational frequencies can be affected by interactions with the phospholipid bilayer and our other molecules. Variations in C=C vibrations reveal information about the insertion and orientation of these compounds within the phytosome. Stability and Functionality: Changes in vibrational modes can reflect the stability and functionality of nanophytosomes. For instance, variations in -OH vibrations indicate alterations in hydration and hydrogen bonding networks, affecting the stability and release of encapsulated compounds (Fathi et al., 2023; Nsairat et al., 2023). Changes in C=C vibrations provide insight into bilayer packing and fluidity, impacting permeability and cellular uptake (Harwansh et al., 2024; Lukhele et al., 2023).

Overall, the interactions between catechin, essential oils, and the phospholipid bilayer involve complex electrostatic, hydrophobic, and hydrogen bonding forces. These interactions modify the surface charge, fluidity, and stability of nanophytosomes, ultimately influencing their functionality. FTIR techniques can offer valuable insights into these molecular interactions by analyzing changes in vibrational frequencies.

***Encapsulation characteristics:*** In general, the encapsulation efficiency (EE) and loading capacity (LC) of nanophytosomes depend on the size and polarity of the bioactive ingredient (Taghizadeh et al., 2023). The EE and LC values for C-NPs containing 2.5, 5, or 10 mg of catechin were 87 %, 90 %, and 93 % (EE) and 70 %, 73 %, and 78 % (LC), respectively, whereas for the PEO-NPs containing 10, 20, or 30 mg of essential oil they were 75 %, 80 %, and 84 % (EE) and 62 %, 68 %, and 74 % (LC), respectively (see Table 1). As the concentration of the active ingredient increased, the EE and LC values also increased, which suggests that the nanophytosomes contained sufficient regions within them for encapsulation of the bioactives. Other researchers have also reported relatively high EE and LC values for bioactives in nanophytosomes, including cumin essential oil (Khosh Manzar et al., 2021a; Khosh Manzar et al., 2021b), epigallocatechin gallate (Zou et al., 2014), and rutin (Afshin Babazadeh et al., 2017). Previous researchers have reported that various factors may impact the encapsulation properties of nanophytosomes, including phospholipid properties, membrane stiffness, preparation method, and water content (Ahmad et al., 2019; Ghorbanzade et al., 2017).

***Turbidity:*** The turbidity of delivery systems can impact their application in different kinds of food and beverage products, which may be clear, cloudy, or opaque. Generally, the turbidity of a colloidal dispersion depends on the particle size, concentration, and refractive index (Amiri et al., 2018; Patil & Jadhav, 2014). The turbidity values for all the samples were below 0.15 cm^−1^, indicating that they were only slightly cloudy. The turbidity decreased slightly by increasing catechin or PEO concentration, which can be attributed to the decrease in particle size, as this would lead to weaker light scattering. Other researchers have also reported that bioactive-loaded nanophytosomes can be produced that have a relatively low turbidity (Amiri et al., 2018; Khosh Manzar et al., 2021a; Khosh Manzar et al., 2021b).

**Fig. 1:**
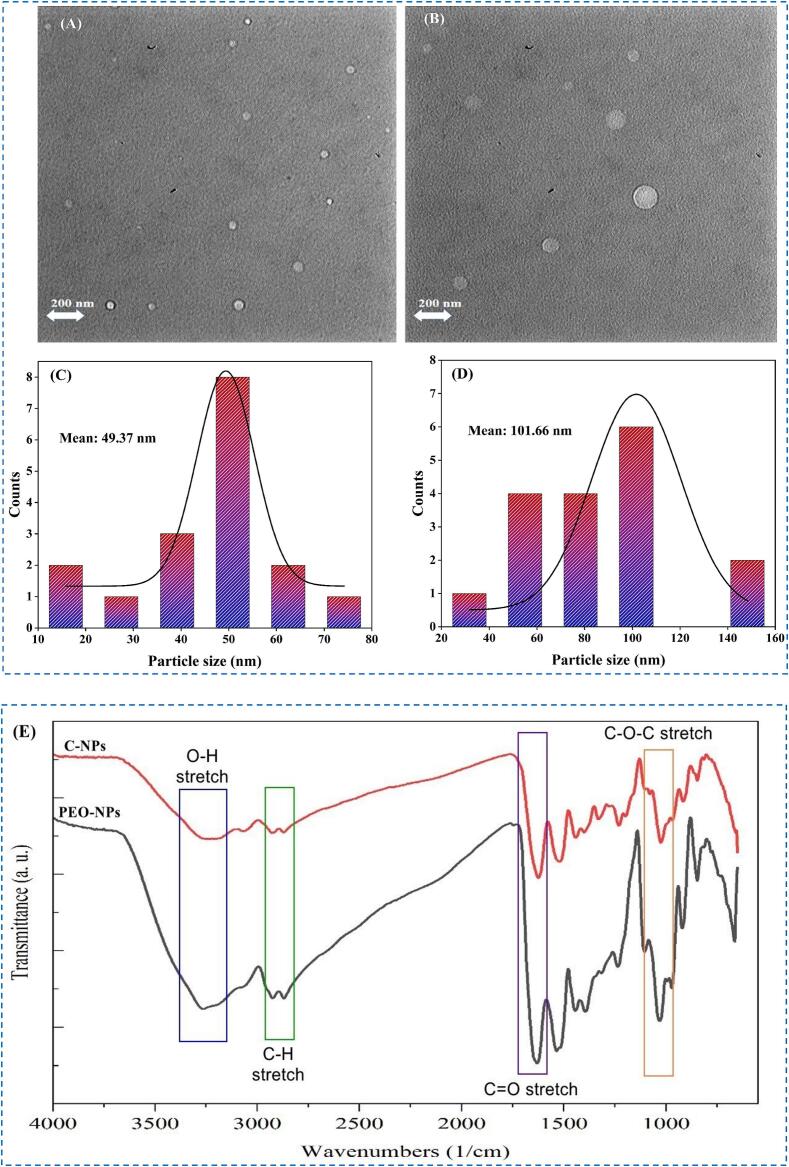
Transmission electron micrograph (TEM) of PEO-NP (A) and C-NP (B); Size distribution of PEO-NP (C) and C-NP (D); and Fourier transform infrared spectroscopy (FT-IR) of C-NP and PEO-NP treatments (E). Catechin nanophytosome (C-NP), *Perovskia abrotanoides* essential oil (PEO) nanophytosome (PEO-NP).

***Microstructure***: Transmission electron microscopy (TEM) was used to provide information about the structure of the nanophytosomes (Wang et al., 2011). The TEM images show that the particles in the C-NP and PEO-NP dispersions were relatively small, spherical, and evenly dispersed throughout the system (Fig. 1**, A** and **B**). The size distribution for C-NP and PEO-NP was found to be 101.66 nm and 49.37 nm, respectively (Fig. 1**, C** and **D**).

***FTIR analysis***: Fourier-transform infrared spectroscopy (FTIR) was used to provide some insights into the composition and molecular interactions within the nanophytosomes (Fig. 1**E**), due to its ability to provide information about the nature of the functional groups present (Ghanbarzadeh et al., 2016a; Ghanbarzadeh et al., 2016b). The spectra of the C-NP and PEO-NP samples contained evidence of the peaks normally seen for lecithin. For example, lecithin contains peaks at 3395 cm^−1^ due to stretching of free OH groups of alcohol esters, at 2925–2856 cm^−1^ due to stretching of alkane groups (CH_2_), at 1735 cm^−1^ due to vibration of carbonyl groups (C = O), and at 580 cm^−1^ due to PO stretching of phosphate groups (Khosh manzar et al., 2021). The FTIR spectra of the C-NP and PEO-NP samples also exhibited distinct peaks at 3300 cm^−1^, which correspond to -OH stretching vibrations, and at 1363 cm^−1^ which correspond to -OH plane bending vibrations (Fig. 1 **E**) (Liu et al., 2014). The characteristic peaks between 1400 and 1600 cm^−1^ were attributed to the C=C stretching vibration of the aromatic ring, and the peak between 1200 and 1300 cm^−1^ was attributed to the C-O/C-C stretching vibration, which provide evidence of the presence of the bioactive compounds (Jiang et al., 2021; Ramos-Tejada et al., 2002). The peak at 3387 cm^−1^ is related to the O—H stretching vibration, the peak at 2929 cm^−1^ is related to the C—H stretching vibration, the peak at 1652 cm^−1^ is related to H-O-H bending vibration, the peak at 1155 cm^−1^ is related to C—O stretching, and the peak at 1028 cm^−1^ is related to C-O-C stretching vibration (Wang et al., 2011). The fingerprint regions observed in the case of catechin at 3479, 1653, 1283, 1144, and 1070 cm^−1^ correspond to the presence of the aromatic quarter ring, the OH shift of the aromatic alcohol, the CO stretch of the aromatic alcohol and aliphatic secondary alcohol, and the aliphatic secondary alcohol, respectively (Ahmad et al., 2019).

***Antioxidant activity***: No significant differences were observed between the antioxidant activities of the free bioactives and the bioactive-loaded nanophytosomes on 0 day (Table 2), indicating that the encapsulation method did not adversely affect the antioxidant activity of the bioactives. Over time, the antioxidant activity of all treatments decreased, which can be attributed to their chemical degradation when exposed to air (Ho et al., 2017). However, this decrease was less for the bioactive-loaded nanophytosomes than for the free bioactives, indicating the protective effects of encapsulation within the nanophytosomes. These results are consistent with previous studies using phytosomes loaded with turmeric (Karimi et al., 2018), cumin essential oil (Khosh Manzar et al., 2021a; Khosh Manzar et al., 2021b), or rutin (Afshin Babazadeh et al., 2017). After 30 days, the antioxidant activity of both types of nanophytosomes increased with increasing bioactive concentration (Table 2), which would be expected because there were more antioxidant molecules available inside the delivery systems.

**Table 2:** Antioxidant activity and antimicrobial (diameter of inhibition zone) activity of C-NP and PEO-NP treatments.

Antimicrobial (mm)	Antioxidant (%)	Treatments			
*B. subtilis*	*S. aureus*	*E. coli*	30th day	1th day	
8.5 ± 0.14^b^	8 ± 0.17^b^	11 ± 0.18^c^	57 ± 3.4^c^	82 ± 7.7^b^	C-NP-1
8 ± 0.15^b^	8 ± 0.15^b^	12.5 ± 0.24^a^	60 ± 4.2^b^	83 ± 8.1^ab^	C-NP-2
9 ± 0.17^d^	9 ± 0.18^d^	13 ± 0.27^a^	63 ± 5.6 ^a^	83 ± 7.9^ab^	C-NP-3
7 ± 0.11^c^	7.5 ± 0.13^c^	9 ± 0.16^d^	37 ± 4.4^d^	84 ± 7.8 ^a^	Catechin
9 ± 0.13^b^	10.5 ± 0.21^b^	13 ± 0.19^a^	38 ± 4.8^c^	71 ± 5.5^c^	PEO-NP-1
11 ± 0.17^c^	11 ± 0.22^c^	14 ± 0.25^a^	41 ± 3.8^b^	71 ± 6.1^c^	PEO-NP-2
11.5 ± 0.19^a^	12 ± 0.24^a^	14.5 ± 0.28^d^	45 ± 4.3 ^a^	73 ± 7.3^ab^	PEO-NP-3
8 ± 0.11^b^	8.5 ± 0.12^b^	10 ± 0.17^c^	24 ± 2.0^d^	74 ± 6.0 ^a^	PEO

Nanophytosome (NPs), Catechin nanophytosome (C-NP), *Perovskia abrotanoides* essential oil (PEO) nanophytosome (PEO-NP).

***Antibacterial activity***: Previous researchers have reported that both catechin and PEO exhibit antimicrobial activities (Aree & Jongrungruangchok, 2016; Ghaffari et al., 2018). As shown in Table 2, all treatments had an antimicrobial effect on the bacteria used in this study. However, the bioactive-loaded nanophytosomes showed greater antibacterial effects than the free bioactives. Moreover, the antimicrobial activity increased with increasing bioactive concentration, as would be expected. These results are consistent with those of previous studies on nanophytosomes containing garlic essential oil (Ghaffari et al., 2018) and thyme essential oil (Moghimi et al., 2016). Phytosomes were effective against both bacteria, although their effect on *E. coli* was somewhat greater than on Gram-positive bacteria (*B. subtilis* and *S. aureus*), which is attributed to differences in the cell wall structure of these bacteria (Alizadeh-Sani et al., 2019; Ashraf et al., 2013; Azizi-Lalabadi et al., 2019). Gram-positive bacteria have a thick peptidoglycan layer, which may better protect them, versus Gram-negative ones (AArabi et al., 2017).

***Gastrointestinal stability***: Catechin and PEOs are prone to oxidation due to the presence of hydroxyl groups in phenol rings (Rashidinejad et al., 2021). The stability of these two bioactive substances was therefore measured under simulated gastric and small intestinal conditions (Table 3).

**Table 3:** Characterization of in vitro release from C-NP and PEO-NP treatments.

	Time (h)	0.5	1	1.5	2	2.5	3	3.5	4
SGF	PEO	97 ± 7.2^ab^	93 ± 4.7^c^	89 ± 8.4^c^	85 ± 7.4^c^	–	–	–	–
	PEO-NP	98 ± 8.1^a^	97 ± 5.8^a^	94 ± 8.7^a^	91 ± 8.3^a^	–	–	–	–
	Catechin	96 ± 8.3^c^	93 ± 7.8^c^	87 ± 6.7^d^	83 ± 7.2^d^	–	–	–	–
	C-NP	97 ± 6.7^ab^	95 ± 7.4^b^	92 ± 7.3^ab^	90 ± 7.4^ab^	–	–	–	–
SIF	PEO	92 ± 5.5^a^	74 ± 6.2^c^	62 ± 3.7^c^	48 ± 3.1^d^	34 ± 2.7^c^	22 ± 2.7^c^	15 ± 1.2^c^	8 ± 0.9^c^
	PEO-NP	88 ± 5.4^c^	75 ± 5.4^c^	59 ± 3.4^d^	50 ± 4.3^c^	38 ± 2.8^b^	30 ± 2.8^a^	24 ± 2.1^a^	17 ± 1.3^a^
	Catechin	90 ± 4.6^b^	83 ± 6.7^a^	70 ± 4.5^a^	57 ± 4.7^a^	45 ± 3.1^a^	25 ± 2.5^b^	13 ± 1.1^d^	6 ± 0.7^d^
	C-NP	87 ± 5.5^c^	80 ± 6.9^b^	67 ± 4.1^b^	54 ± 4.3^b^	32 ± 2.5^d^	24 ± 2.3^b^	19 ± 2.2^b^	15 ± 1.1^b^

Nanophytosome (NPs), Catechin nanophytosome (C-NP), *Perovskia abrotanoides* essential oil (PEO) nanophytosome (PEO-NP), Simulated gastric fluids (SGFs), Simulated intestinal fluids (SIFs).

The nanophytosomes had a significant protective effect on the catechin and essential oil under simulated gastrointestinal conditions (*p < 0.05*). For simulated gastric conditions, there was only a slight decrease in the concentration of catechin and essential oil present over time, which can be attributed to their relatively high resistance to acidic conditions. Even so, the rate of degradation of the two bioactives was slower when they were encapsulated within the nanophytosomes than when they were in the free form, which indicates that encapsulation improved their gastric stability. For instance, over 90 % of both bioactives remained after 2 h of incubation under gastric conditions, whereas only 85 % of free essential oil and 83 % of free catechin remained. For simulated small intestine conditions, where there was a steep decline in catechin and essential oil concentration over time, which can be attributed to the susceptibility of these phenolic substances to alkaline conditions. Again, encapsulation greatly improved the resistance of the bioactives to gastrointestinal conditions. For instance, around 8 % and 17 % of the free and encapsulated essential oil remained after four hours, whereas 6 % and 15 % of the free and encapsulated catechin remained. Other researchers have also reported that other phytochemicals (such as green tea extract) are more stable under stomach than small intestine conditions (Zou et al., 2014), and that encapsulation in nanophytosomes can improve their resistance to gastrointestinal degradation (Nazari, Ghanbarzadeh, Kafil, et al., 2019).

***Physical stability***: The physical stability of delivery systems during storage is critical for their effectiveness in practical applications. For this reason, changes in the particle characteristics of the phytosomes were measured over time (Table 4). Both C-NP and PEO-NP dispersions remained relatively stable during storage, with only a small rise in mean particle diameter being observed after 30 days. There was also no significant change in the polydispersity index values and little change in the zeta potential values during storage. This result suggests that the nanophytosomes were relatively resistant to particle aggregation and gravitational separation. The good resistance to aggregation may have been due to the strong electrostatic repulsion between the nanophytosomes, arising from their relatively high negative charges. The good resistance to gravitational separation may have been due to their relatively small particle sizes. The EE and LC values decreased slightly during storage, which may have been due to some degradation or leakage of the bioactive compounds over time (Sahari et al., 2016).

**Table 4:** Physical stability of C-NP and PEO-NP treatments during storage period.

LC											
(%)	EE										
(%)	Zeta Potential (mV)	PDI	Z average								
(nm)	Treatments										
30th day	1th day	30th day	1th day	30th day	1th day	30th day	1th day	30th day	1th day		
65 ± 1^c^	70 ± 2^c^	86 ± 1^bc^	87 ± 1.5^bc^	−46 ± 3.4^a^	−49 ± 4.4 ^a^	0.20 ± 0.04 ^a^	0.20 ± 0.07 ^a^	193 ± 11^a^	181 ± 12^a^	C-NP-1	**C-NP**
67 ± 1^b^	73 ± 2^b^	87 ± 3^ab^	90 ± 2^ab^	−46 ± 2.4^a^	−48 ± 5.3 ^a^	0.20 ± 0.03 ^a^	0.20 ± 0.08 ^a^	189 ± 10^ab^	178 ± 12^ab^	C-NP-2	
70 ± 2^a^	78 ± 3^a^	88 ± 1.5^a^	93 ± 1^a^	−45 ± 2.7^b^	−45 ± 5.7 ^b^	0.19 ± 0.02 ^b^	0.19 ± 0.05 ^b^	176 ± 11^c^	162 ± 1 ^c^	C-NP-3	
56 ± 2^c^	62 ± 1^c^	71 ± 3^c^	75 ± 2^c^	−50 ± 5.4a	−52 ± 4.9 ^a^	0.19 ± 0.06 ^a^	0.18 ± 0.06^a^	174 ± 8^a^	166 ± 11 ^a^	PEO-NP-1	**PEO-NP**
65 ± 2^b^	68 ± 1^b^	76 ± 2^b^	80 ± 1^ab^	−47 ± 3.8^b^	−47 ± 4.4 ^b^	0.18 ± 0.03 ^b^	0.17 ± 0.05 ^b^	165 ± 9^b^	153 ± 12 ^b^	PEO-NP-2	
69 ± 1^a^	74 ± 3^a^	79 ± 1^a^	84 ± 1.5^a^	−32 ± 2.1^c^	−34 ± 3.8 ^c^	0.17 ± 0.03 ^c^	0.17 ± 0.05 ^b^	153 ± 8^c^	140 ± 10^c^	PEO-NP-3	

PDI; Polydisperse index; EE: Encapsulation efficacy; LC: Loading capacity, Nanophytosome (NP), Catechin nanophytosome (C-NP), *Perovskia abrotanoides* essential oil (PEO) nanophytosome (PEO-NP).

### 3.2 Application of active packaging films on rainbow trout preservation

In this series of experiments, the ability of nanophytosome-loaded packaging films **(Fig. S1**) to extend the shelf life of fish fillets was examined using both chemical and microbiological analysis. Nanophytosomes containing 10 mg of catechin (C-NP-3, T1), and 30 mg of PEO (PEO-NP-3, T2) were used in these studies.

#### 3.2.1 Chemical analysis

***pH:*** Initially, the samples have a pH of around 6.64. During storage, the pH first decreased but then it increased (Table 5). The initial decrease was attributed to glycolysis, whereas the later increase was attributed to the metabolic action of the bacterial enzymes (Zhang et al., 2019). The final pH values for the control treatments, catechin-loaded nanophytosomes (T1), and essential oil-loaded nanophytosomes (T2), were 7.8, 6.9, and 7.2, respectively (see Table 5).

**Table 5:** Chemical and microbial changes of rainbow trout samples during storage time at 4 °C.

Psychrotrophic (log CFU/g)	Total count (log CFU/g)	TVB-N (mg N/100 g)	TBA (mgMDA/kg)	pH	Treatments	Day
3.8 ± 0.012 ^a^	3.03 ± 0.014 ^a^	14.4 ± 1.2 ^a^	0.13 ± 0.002 ^a^	6.6 ± 0.1 ^a^	C	0
3.8 ± 0.017 ^a^	3.03 ± 0.015 ^a^	14.4 ± 1.3 ^a^	0.13 ± 0.005 ^a^	6.6 ± 0.1 ^a^	T_1_	
3.8 ± 0.022 ^a^	3.03 ± 0.014 ^a^	14.4 ± 1.1 ^a^	0.13 ± 0.005 ^a^	6.6 ± 0.1 ^a^	T_2_	
4.8 ± 0.034 ^a^	4.89 ± 0.07 ^a^	18.9 ± 2.2 ^a^	0.27 ± 0.007 ^b^	6.5 ± 0.1 ^a^	C	3
4.25 ± 0.063 ^b^	3.27 ± 0.07 ^c^	15.7 ± 2.4 ^c^	0.22 ± 0.007 ^c^	6.5 ± 0.1 ^a^	T_1_	
4.59 ± 0.034 ^b^	3.58 ± 0.06 ^b^	17.6 ± 2.7 ^b^	0.35 ± 0.008 ^a^	6.50 ± 0.1 ^a^	T_2_	
6.74 ± 0.088 ^a^	6.56 ± 0.08 ^a^	26.1 ± 2.9 ^a^	0.49 ± 0.008 ^a^	7.0 ± 0.1 ^a^	C	6
4.89 ± 0.064 ^c^	3.87 ± 0.06 ^bc^	17.2b ± 1.45^c^	0.32 ± 0.007 ^b^	6.7 ± 0.11 ^b^	T_1_	
5.27 ± 0.08 ^b^	4.21 ± 0.05 ^b^	19.8 ± 1.56 ^b^	0.49 ± 0.009 ^a^	6.9 ± 0.1 ^a^	T_2_	
7.97 ± 0.09 ^a^	7.21 ± 0.09 ^a^	29.3 ± 2.87 ^a^	0.67 ± 0.01 ^a^	7.4 ± 0.1 ^a^	C	9
5.24 ± 0.067 ^c^	4.07 ± 0.08 ^b^	18.5 ± 2.46 ^c^	0.37 ± 0.008 ^c^	6.8 ± 0.1 ^b^	T_1_	
6.01 ± 0.11 ^b^	4.65 ± 0.05 ^b^	22.7 ± 2.14 ^b^	0.56 ± 0.007 ^b^	6.9 ± 0.1 ^b^	T_2_	
8.43 ± 0.12 ^a^	8.6 ± 0.11 ^a^	33.2 ± 2.68 ^a^	0.84 ± 0.01 ^a^	7.8 ± 0.1 ^a^	C	12
6.1 ± 0.11 ^b^	4.3 ± 0.08 ^b^	19.7 ± 2.56 ^c^	0.43 ± 0.012 ^c^	6.9 ± 0.1 ^b^	T_1_	
6.5 ± 0.09 ^b^	4.9 ± 0.09 ^b^	24.3 ± 2.87 ^b^	0.64 ± 0.013 ^b^	7.2 ± 0.1 ^b^	T_2_	
Catechin nanophytosome (T1): Essential Oil nanophytosome (T2); Control (C).						

***TBA:*** The initial TBA content of the fresh fish was 0.13 mg MDA/kg (Table 5), which is well below the levels where an off odor is detectable (> 1–2 mgMDA/kg) and where the fish would be considered to be unsafe for consumption (> 5 mgMDA/kg) (Alizadeh-Sani et al., 2020; Yin et al., 2022). As expected, the TBA levels increased over time for all treatments, with the control group exhibiting the highest rise (to 0.84 mgMDA/kg). The greater resistance of the samples containing the nanophytosomes to oxidation was attributed to the antioxidant activity of the catechin and PEOs. At the end of storage, the packaging films containing catechin-loaded nanophytosomes resulted in lower TBA levels (0.43 mgMDA/kg) than those containing essential oil-loaded nanophytosomes (0.64 mgMDA/kg), which suggests that the catechin had a higher antioxidant activity. Other researchers have also reported that catechin-loaded nanocapsules exhibit good antioxidant properties (Li et al., 2018).

***TVB-N:*** The TVB-N value provides a measure of the deterioration of fish during storage, as these substances are a result of the chemical degradation of proteins. The initial TVB-N value of the raw fish was 14.4 mg N/100 g (see Table 5). This value increased over time for all treatments, reaching 32.2, 19.7, and 24.3 mg N/100 g for the control, catechin-loaded nanophytosomes (T1), and essential oil-loaded nanophytosomes (T2), respectively. The lower levels in the T1 and T2 groups can be attributed to the antioxidant and antibacterial properties of the bioactive-loaded nanophytosomes (Alizadeh-Sani et al., 2020; Yin et al., 2022). The fact that the TVB-N value was lower for T1 than T2 suggests that the catechin was a more potent antioxidant and/or antimicrobial than the essential oil.

#### 3.2.2 Microbial analysis

***TVC:*** The initial total viable count (TVC) of the fresh fish samples was 3.03 log CFU/g (Table 5), which is consistent with previous findings (Alizadeh-Sani et al., 2020; Yin et al., 2022). At the end of the study, the control sample had a TVC value of 8.6 log CFU/g, while the T1 and T2 samples had values of 4.3 and 4.9 log CFU/g, respectively. These results indicate that the catechin-loaded nanophytosomes had stronger antimicrobial properties than the essential oil-loaded nanophytosomes, which is consistent with previous observations (Khosh Manzar et al., 2021a; Khosh Manzar et al., 2021b). In previous studies, researchers have reported that the shelf life of shrimp can be enhanced using cinnamon-loaded nanophytosomes (Nazari, Majdi, Milani, et al., 2019) or nisin-loaded nanoliposomes (Pouryousef et al., 2022). Moreover, catechin nanoparticles have been shown to exhibit antimicrobial effects against various bacteria, including *Escherichia coli, Staphylococcus aureus,* and *Bacillus subtilis* (Li et al., 2018). Thus, the ability of the bioactive-loaded nanophytosomes to reduce the TVC values in our study can be attributed to their good antibacterial effects.

***PBC:*** The initial psychrotrophic bacteria count was 3.8 log CFU/g (Table 5). Over time, the control sample exhibited the highest increase, reaching 8.43 log CFU/g at the end of the study. Treatments T1 and T2 showed lower growth rates, reaching 6.1 and 6.5 log CFU/g, respectively. These changes show the inhibitory and antimicrobial effects of essential oil and catechin compounds (Gál et al., 2023; Veiko et al., 2023).

## 4 Conclusion

Nanophytosomes are being explored as a means of encapsulating, protecting, and delivering natural preservatives in food and beverage applications. This study demonstrates the successful development and application of nanophytosomes loaded with Perovskia abrotanoides essential oil and catechin as innovative components of active packaging films for the preservation of refrigerated rainbow trout fillets. The nanophytosomes, prepared via the thin layer hydration method, exhibited desirable physicochemical properties, including small particle sizes (< 200 nm), low polydispersity indices (≤ 0.2), and high encapsulation efficiency, which enhanced the stability and bioactivity of the encapsulated compounds. The incorporation of these bioactive-loaded nanophytosomes into packaging films significantly extended the shelf life of fish fillets by suppressing microbial growth and oxidative deterioration, outperforming free bioactives in antioxidant and antibacterial efficacy. These findings highlight the potential of nanophytosome-functionalized active packaging as a sustainable and effective solution to meet the growing demand for natural, high-performance food preservation technologies. While challenges related to scalability, regulatory approval, and consumer acceptance remain, the promising results of this study cover the way for future advancements in plant-based active packaging systems, offering a transformative approach to enhancing food safety, quality, and sustainability across the food industry.

Here are some potential limitations of the study described in the provided text: *Limited scope of bioactive concentrations:* The study tested specific concentrations of nanophytosomes (PEO-NP at 10, 20, and 30 mg; C-NP at 2.5, 5, and 10 mg), which may not fully represent the optimal range for practical applications. Higher or lower concentrations could yield different effects on particle size, encapsulation efficiency, or bioactivity. *Focus on a single bioactive species:* The research focused on nanophytosomes loaded with Perovskia abrotanoides essential oil and catechin. The findings may not be generalizable to other bioactive compounds, as different plant extracts or bioactives may exhibit varying stability, encapsulation efficiencies, or interactions with packaging materials. *Short-Term storage evaluation*: The study evaluated the stability under refrigerated conditions, but long-term storage effects (beyond the tested period) or performance under varying environmental conditions (e.g., non-refrigerated or fluctuating temperatures) were not addressed, limiting insights into real-world applications. *Scalability and cost constraints*: While the nanophytosomes showed promise, the study did not discuss the feasibility of scaling up the thin layer hydration method for industrial production or the cost-effectiveness of incorporating nanophytosomes into active packaging, which could pose practical limitations. *Regulatory and consumer acceptance:* The study does not address potential regulatory hurdles or consumer acceptance of nanophytosome-based packaging. Safety, toxicity, and public perception of nanomaterials in food packaging remain significant challenges that were not explored. These limitations could be considered to provide a balanced perspective and guide future research.

## CRediT authorship contribution statement

**Mohammad Maleki:** Writing – original draft, Data curation, Conceptualization. **Mahmood Alizadeh Sani:** Writing – original draft, Methodology, Investigation, Conceptualization. **Roya Rezaeian-Doloei:** Writing – original draft, Investigation. **David Julian McClements:** Writing – review & editing, Visualization, Investigation. **Mohammad Mohsenzadeh:** Writing – original draft, Methodology, Investigation, Conceptualization.

## Funding

This research was financially supported by a postdoctoral grant no. FUM-PD-24223 from the Research Council of the Ferdowsi University of Mashhad.

## Declaration of competing interest

The authors declare that they have no known competing financial interests or personal relationships that could have appeared to influence the work reported in this paper.

## Data Availability

Data will be made available on request.

## Appendix A Supplementary data

Supplementary material Image 1
