# Supplementary material for: Nanophytosome-functionalized active packaging films for preservation of refrigerated rainbow trout
Source: Food Chem X. 2025 Jun 28;29:102708. doi: 10.1016/j.fochx.2025.102708 (PMC12268848; doi:10.1016/j.fochx.2025.102708)
Supplement: Supplementary file 1 — Supplementary material [file mmc1.docx]

Supplementary information.

**Nanophytosome-functionalized active packaging films for preservation of refrigerated** **rainbow trout**


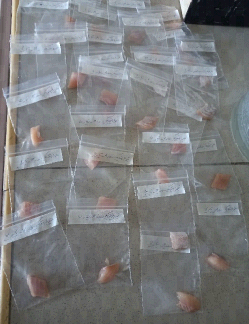

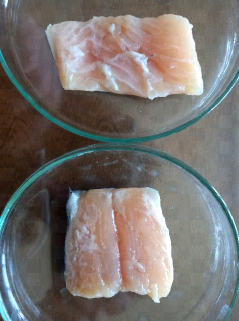


**Figure S1**. Fish samples packaged in active films
